# Working Memory Capacity Depends on Attention Control, but Not Selective Attention

**DOI:** 10.3390/bs13020092

**Published:** 2023-01-22

**Authors:** Alexander I. Kotyusov, Dauren Kasanov, Alexandra I. Kosachenko, Anastasia S. Gashkova, Yuri G. Pavlov, Sergey Malykh

**Affiliations:** 1 Academic and Research Laboratory of Neurotechnology, Ural Federal University, Ekaterinburg 620000, Russia; 2 Center of Population Research, Ural Institute of Humanities, Ural Federal University, Ekaterinburg 620002, Russia; 3 Institute of Medical Psychology and Behavioral Neurobiology, University of Tübingen, 72076 Tübingen, Germany; 4 Developmental Behavioral Genetics Lab, Psychological Institute of Russian Academy of Education, 9-4 Mokhovaya str., Moscow 125009, Russia; 5 Department of Psychology, Lomonosov Moscow State University, 11-5, Mokhovaya Str., Moscow 125009, Russia

**Keywords:** working memory, individual differences, attention control, selective attention

## Abstract

Working memory and attention are interrelated constructs that are sometimes even considered indistinguishable. Since attention is not a uniform construct, it is possible that different types of attention affect working memory capacity differently. To clarify this issue, we investigated the relationship between working memory capacity and various components of attention. The sample consisted of 136 healthy adult participants aged 18 to 37 years (M = 20.58, SD = 2.74). Participants performed tasks typically used to assess working memory (operation span, change detection, simple digit span, and adaptive digit span tasks), selective attention (visual search task), and attention control (Stroop and antisaccade tasks). We tested several models with working memory and attention, either as a unitary factor or being divided into selective attention and attention control factors. A confirmatory factor analysis showed that the model with three latent variables—working memory capacity, attention control, and selective attention—fit the data best. Results showed that working memory and attention are distinct but correlated constructs: working memory capacity was only related to attention control, whereas attention control was related to both constructs. We propose that differences in working memory capacity are determined only by the ability to maintain attention on the task, while differences in the ability to filter out non-salient distractors are not related to working memory capacity.

## 1. Introduction

Information selection and retention of information in an accessible state play an important role in human life because they underlie the mastery of writing, counting, and other learning skills that determine a person’s success. Working memory is referred to as the mechanisms and processes that keep the mental representations most relevant at the present time for the current cognitive task available for processing (e.g., [1,2,3,4]). The phenomenon of “attention” is considered a complex process that includes a plethora of subcomponents, such as orientational and attention control systems [4,5,6]. The function of the orientation system (selective attention) is to select and filter sensory information based on the properties of the stimulus and the correspondence of its features to the task (bottom-up distractor suppression). The attention control supports maintenance of attention on the task being performed and adaptive control (responding to prompts, feedback, and constant behavior correction). To elucidate the processes underlying performance in working memory tasks, it is essential to examine the links between the components of attention and working memory [7]. Despite extensive research, the literature is inconsistent on this matter [8,9,10].

Attention control (aka “cognitive control”), either as a general factor or as a combination of multiple functions, has been associated with working memory capacity [11,12,13,14]. Working memory was associated with components of attention control such as response suppression [8,15], goal maintenance and inhibitory control [9,15,16,17,18], and protection from proactive interference [19]. For example, participants with higher working memory capacity demonstrate higher scores in tasks such as the antisaccade task [16], in which participants must proactively suppress their natural desire to look at the distractor, and Stroop task, where participants respond to the color of the ink rather than the written word, thus engaging inhibitory control [8,17]. The ability to focus on task-relevant information while resisting interference by task-irrelevant information and distraction, as in Stroop and antisaccade tasks, may also contribute to working memory capacity.

The other subcomponent of attention—selective attention—provides filtering of distractors and selection of the target information to be processed and stored in working memory [20,21]. As an example, in the visual search task, two categories of items appear in the visual display, but only one must be amplified in attention and detected, and the other ignored. These functions are often associated with attention control [1,2], but they were also considered a distinct factor contributing to working memory capacity. There are theoretical reasons for looking at selective attention as a separate process that may contribute to working memory function: consolidation in working memory starts only after passing the selection filter [22], so the better the selection, the more relevant information gets into working memory. In accordance with this idea, Peltier and Becker [10] found a correlation between visual search and visual working memory performance. Therefore, selective attention may be considered as one of the target factors that contribute to working memory capacity.

The aim of the present study was to examine how attention control and selective attention are related to working memory capacity and each other. To test the contribution of components of attention (cognitive) control such as proactive and reactive control, we employed three versions of Stroop task with a variable number of incongruent trials; antisaccade task was employed to load on inhibitory control; a visual search task was employed to study selective attention; visual arrays, operation span, and digit span tasks were used to determine working memory capacity.

## 2. Materials and Methods

### 2.1. Participants

We aimed to collect a sample of at least 100 participants (after exclusions) to be able to reliably detect stable medium-size correlations. With the width of the corridor of stability w = 0.15, we would need a sample of 93 participants to detect effect size r = 0.3 with 80% confidence [23]. For comparison, a sensitivity analysis in G*Power would result in 84 participants required to detect effect size of 0.3 with 80% power and alpha level of 0.05.

In total, 136 participants (123 females) were recruited for this study at the Ural Federal University. Most of the participants were university students. Participants were between the ages of 18 and 37 (M = 20.58, SD = 2.74). Each participant received an approximately $6.5 (400 rubles) payment for their participation. The female/male ratio in our sample as well as the level of payment were typical for a department of psychology in Russia. The study protocol was approved by the ethics committee of the Ural Federal University (N 01/27.04.2018) and signed informed consent was obtained from all participants. All participants were native Russian speakers. The participants had a normal or corrected-to-normal vision and no history of neurological or mental diseases. Seventeen participants did not return to the second session and were excluded from all analyses. Three more participants were excluded due to technical errors (one) or did not follow the instruction (two participants) in one or more tasks in the first session. Thus, 116 participants comprised the sample that entered the analyses.

### 2.2. Measures

The study was conducted in two sessions. Each participant was individually tested in the laboratory with the luminance in the room set to 380 lux. In the first session, the participants signed the informed consent and completed several cognitive tasks in random order: Stroop, verbal operation span, visual arrays (also known as change detection), simple digit span, visual search, and antisaccade task. In the second session, participants completed the adaptive digit span task to determine their verbal working memory capacity more precisely. None of the tasks included accuracy feedback after practice trials. Background color for all tasks was gray (RGB: 128, 128, 128). For the text stimuli, white (RGB: 256, 256, 256) “Arial” font type was used.

In the first session, the tasks were administered on the laptop with 15.6” screen size. Participants sat at a distance of 52 cm from the screen with the experimenter sitting about two meters away. In the second session, the participants were sitting in a room alone in front of a 23.5” screen. The distance to the screen was 80 cm.

The average duration of the first session was 1.5 h, and the duration of the second session varied between one and 2.5 h.

#### 2.2.1. Attention Control Tasks

##### Stroop Task

We ran three series of the classic Stroop task. Participants were presented with a colored word (“blue”, “green”, “red”, or “yellow”) which had either of four different colors: blue, green, red, or yellow. Letter size was 2.2 visual degrees. The participants had to indicate which color the word was by pressing the keys on the keyboard (1—red, 2—green, 9—blue, 0—yellow). At the bottom of the screen, brief instructions were displayed during all trials. Congruent trials included words matched to color, i.e., green printed in green color. Incongruent trials included words printed in different colors (e.g., green printed in blue). Each stimulus was shown until the participant responded. The next stimulus was presented half a second after the response.

The block with the standard version of the Stroop task included 50% congruent and 50% incongruent trials. The proactive control block was made to activate proactive cognitive control and included 75% incongruent and 25% congruent trials. The reactive control block was supposed to activate reactive control and included 25% incongruent and 75% congruent trials. The total number of trials was 48 per block. Before the task, the participants completed a training block, which included 16 trials of the standard version of the task. The dependent variable was the Stroop effect, defined as the difference between reaction time in congruent and incongruent trials. The average time to complete all versions of the task was seven minutes.

##### Antisaccade Task

The antisaccade task is considered a test of inhibitory control [24,25]. We used the behavioral version of the antisaccade task in which participants had to suppress prosaccade and move their gaze in the opposite direction. Instruction font size was 0.44 visual degrees. The test started with a fixation cross presented in the center of the screen for 2000–3000 ms. Then, the asterisk symbol (*) was presented for 300 ms at a 12.3° visual angle to the left or right of the central fixation cross. Immediately after that, the target letter “Q” or “O” was presented on the opposite side for 100 ms, and then the positions of the letter and the asterisk were replaced by the symbol ## for 500 ms. Letter size was 1.1 visual degrees. The participant’s goal was to ignore the asterisk and move the gaze to the opposite side and give an answer to which letter was presented by pressing the “O” or “Q” key. The time to respond was not limited. Prior to the main task, participants completed four training trials with feedback. There were 72 trials total in the main task. The average task duration was nine minutes. The dependent variables were accuracy calculated as the proportion of correct trials and the reaction time.

#### 2.2.2. Selective Attention Task

##### Visual Search

Visual search is a key paradigm in studies of selective attention [26]. Participants were instructed to answer if the target letter was presented among distractors or not. Instruction’s text size was 0.55 visual degree. The target letter was “L”, and the distractors were “T”. Letter size was 1.1 visual degrees. Target and distractor letters were positioned and oriented randomly but did not overlap. The set size was 5 letters (i.e., target and four or five distractors), 10, 15, and 20 letters; 25% of the trials included only distractors, and 75% of the trials included both target and distractors. The stimuli were presented for an unlimited time until the participant responded, and the next stimulus appeared immediately after the response. In total, there were 32 trials for each set size. Before the main task, participants completed eight training trials with feedback. The average task time was five minutes. The dependent variables were average reaction time across all set sizes and the difference between reaction time in trials with set size 5 and set size 20. The difference in reaction time between high and low set-size represents the slope of the response, which has previously been reported as a visual search metric [27].

#### 2.2.3. Working Memory Capacity Tasks

##### Visual Arrays Task

In each trial of the visual arrays task, participants were presented with a visual array of colored squares, and after a short retention interval, the second visual array was presented where one of the squares had changed the color or all colors remained the same [28]. Instruction’s font size was 1 visual degree. The size of each square was 1 visual degree. The color set for squares was obtained by combination of maximal and minimal values of RGB scale (e.g., 255,255,0 for yellow) and included black, white, blue, green, red, cyan, purple, and yellow. The first arrays were presented for 500 ms, the delay period was 1000 ms, and the presentation of the second array was presented for 500 ms. As soon as the second array disappeared, the participant had to press the right arrow key if the arrays were the same and the left arrow if they were different. Arrays consisted of two, four, and six colored squares. Each set size was presented 50 times; half of all trials included the same arrays, and half included different arrays. Dependent variables were reaction time and storage capacity, which was calculated according to Saults and Cowan [29] as k_c_ = N × (p(H) − p(FA)), where N is the number of array items, p(H) is the proportion of trials with hits and p(FA) is the proportion of trials with false alarms. We used average k_c_ = (k_c_2 + k_c_4 + k_c_6)/3, as in other studies of individual differences in working memory capacity (e.g., [30]). The average task time was five minutes.

##### Operation Span Task

Each trial included a list of Russian letters consequently presented with a simple arithmetic equation (i.e., 5 × (3 − 1) = 12) presented after each letter. Font size of the letters and operations was 1.1 visual degrees. The duration of each letter presentation was 800 ms. Equations were presented until solved. The sequence length ranged from three to nine letters. Participants were instructed to determine if the equation was correct or not by pressing the right arrow key for “correct” and the left arrow key for “incorrect” answers, after which the letter to remember was presented. After the presentation of the sequence, participants were asked to type in memorized letters in the order in which they were presented. There were two blocks, each included seven trials with different sequence lengths. Based on Conway et al. [31], the dependent variable was the partial span score, calculated as the percentage of letters reported on correct positions. The average task time was seven minutes.

##### Simple Digit Span

In this task, participants were presented with sequences of digits to be remembered and recalled in the correct order. Font size was 1.1 visual degrees. Digits were presented for 500 ms with stimulus onset asynchrony of 2000 ms. The last digit presentation was followed by a retention interval of 1500 ms. If the participant recalled the sequence correctly, the next sequence length increased by one digit. The inability to recall three sequences of the same length ended the task. The dependent variable—digit span—was the maximal length of the sequence that was correctly recalled. The average task time was three minutes.

##### Adaptive Digit Span

A modified version of the simple digit span, the adaptive digit span evaluates verbal working memory capacity more precisely. In each trial, participants were presented with a sequence of digits to be remembered and recalled, similar to the simple digit span. The trial began with an exclamation mark for 0.5 s, followed by a fixation cross for 2 s, and then digits were presented for 0.5 s with SOA of 2 s alternated with the fixation cross. After the presentation of the last digit, the retention interval continued for 3.5 s. After that, the participant had to type in the sequence in the correct order. The response time was unlimited. Font size was 1.1 visual degrees. A successful trial was defined as recollecting all digits in the correct order. The trials were grouped into blocks, each consisting of 10 trials. All sequences in each block were of the same length. If a participant was able to recall seven trials or more in a block, the sequences in the next block were longer by one digit. If the participant recalled fewer than seven trials, the next block consisted of sequences shorter by one digit. The task was finished when the participant completed six blocks consisting of adjacent sequences of each type (i.e., at least 60 trials with <70% accuracy and 60 trials with ≥70% accuracy). Sequence length corresponding to the ≥70% accuracy blocks was considered the individual capacity of verbal working memory and was taken as the dependent variable. As the task was adaptive, participants could complete more trials than the minimum of 120, which, along with self-paced breaks between blocks, contributed to the above-mentioned variability in the task duration. On average, 66 trials in the blocks with ≥70% accuracy and 66 trials in the blocks with <70% accuracy were included in the analysis.

### 2.3. Statistics

The internal consistency of the tasks was assessed using split-half reliability [32] with the Spearman–Brown coefficient [33]. The reliability of the difference measures such as the Stroop effect and the difference in reaction time in the visual search task were assessed by adjusted Cronbach’s alpha [34].

Outlier identification was carried out using the Rosner test [35].

Correlation analysis was performed using the Pearson’s correlation coefficient, and Holm’s correction was used to correct for multiple comparisons [36].

Exploratory factor analysis was performed using the “psych” package from R, orthogonal rotation, and the maximum likelihood analysis method.

We conducted latent variables model estimation using the R lavaan package [37]. Following the work of Hu and Bentler [38], we used several statistical criteria to test whether our model fit the data: the *χ^2^* fit index stating that a smaller nonsignificant value indicates a good fit; Bender’s Comparative Fit Index (CFI) and the non normed fit index (NNFI) stating that a good fit must be >0.90 with desired value >0.95; the standardized root-mean-square residual (SRMR) and the root mean square error of approximation (RMSEA) state that must be <0.08 for a good fit; Akaike information criterion (AIC) and Bayesian information criterion (BIC) were used to examine the relative fit of models.

## 3. Results

### 3.1. Descriptive Statistics

First, we tested the reliability of the measurements and computed correlations between the tasks. The results of the descriptive analysis are presented in Table 1. All tasks showed acceptable levels of reliability, with a reliability coefficient in the 0.65–0.99 range, except for the Stroop effect. Values of skewness and kurtosis coefficients calculated for the entire sample were greater than the critical values for some tasks (the critical value of the skewness coefficient is 2, and the critical value of the kurtosis coefficient is 4; West et al. [39]). After outliers were removed, the values for all tests were in an acceptable range. This sample was used in the subsequent analyses.

Unlike other variables, the Stroop effect for the reactive control version and the proactive control version did not significantly correlate with any of the variables (Table 2). These variables were excluded from further analyses due to the lack of significance in determining individual differences.

### 3.2. Exploratory Factor Analysis

To explore the data, we conducted an exploratory factor analysis. The adequacy was supported by Bartlett’s sphericity test with *χ*^2^ = 233.76, df = 55, *p* < 0.001, and mediocre, but sufficient Kaiser–Meyer–Olkin measure (0.63). We used parallel analysis [40] and the Scree test [41] to determine the optimal number of factors. On the basis of both tests, the best solution was three factors. Factor loadings are presented in Table 3. The model accounted for 53.50% of the variance.

As expected, simple digit span, adaptive digit span, and operation span loaded on a single factor, which can be interpreted as a working memory capacity factor. At the same time, the load of the change detection task was distributed over several factors, with the maximum load on the factor with the Stroop effect. Both scores on the visual search test were assigned to a separate factor. This factor can be interpreted as selective attention. Instead of the expected separate attention control factor, antisaccade accuracy loaded on the working memory factor. Additionally, loads of this variable on other factors were also non-zero.

### 3.3. Confirmatory Factor Analysis

We tested several structural equation models to define which structure of latent variables better explained the data and how different variables related to working memory.

Based on theoretical assumptions and the results of previous empirical studies, several structural models were proposed. In all models, a separate variable was designated as the capacity of working memory. We suggest that it reflects a general factor associated with the ability to retain information, similar to that proposed previously [42]. Other factors were cognitive control and selective attention.

As described above, several hypotheses explain individual differences in working memory. According to the first approach, they stem from the general factor of attention, on which the first model is based. Another approach suggests that individual differences may be due to two factors: selective attention and control of attention. In the second model, the effect of the Stroop task and performance in the antisaccade task were attributed to the attention control factor, similar to the model by Miyake and Friedman [43]. The third model was created based on the assumption that the change detection test reflects the attention control ability rather than working memory [30]. In the fourth model, we hypothesized that the Stroop effect in a test with 50% congruent stimuli could be related to both attention control and selective attention. The performance of the models is presented in Table 4. In addition, the model was tested on the distribution of variables by factors obtained as a result of exploratory factor analysis.

**Figure 1:**
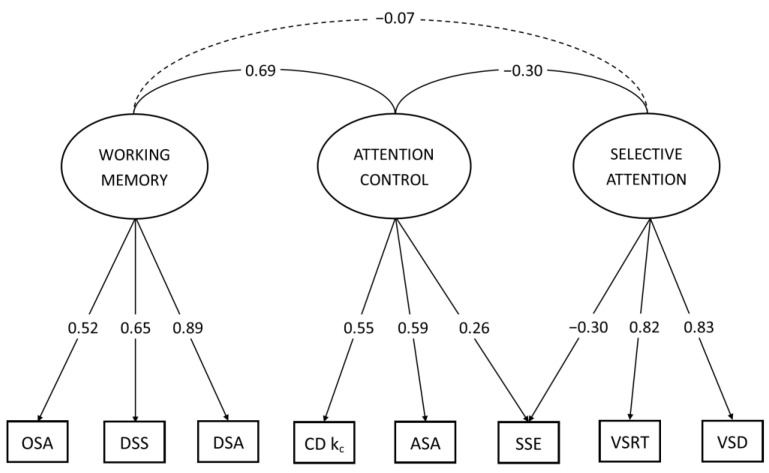
The diagram illustrates a model (Model 4) of WM and the interaction of the attentional components with the best indices. Here, the factor of WM includes OSA (operation span accuracy), DSS (digit span simple), and DSA (digit span adaptive). The attention control factor consists of CD k_c_ (change detection k_c_ average), ASA (antisaccades accuracy), attention control and selective attention share, and SSE (standard Stroop effect). Selective attention comprises VSRT (visual search reaction time) and VSD (visual search difference).

Model 1, which included the general attention factor, showed an unsatisfactory degree of model convergence and was rejected. In other words, the data cannot be described using a general attention factor. The model based on the factors obtained during exploratory analysis did not meet the convergence criteria either; the RMSEA and SRMR fitness indicators were significantly higher than the baseline level of 0.05. Thus, this model had to be rejected. As a result, Models 3 and 4 met the criteria, while the quality indicators of Model 4 were significantly higher than those of Model 3, which allows us to conclude that for a better description of the data, it is necessary to consider the contribution of the Stroop task to both the factor of attention control and selective attention. The final model is shown in Figure 1.

## 4. Discussion

The present study aimed to test whether different types of attention could explain individual differences in working memory capacity. The resulting model demonstrated the presence of two factors: selective attention and attention control. Differences in working memory capacity were explained only by attention control, while selective attention was associated with attention control but not working memory. Thus, first, attention in the context of working memory research cannot be seen as a single factor. Second, the attention control factor is more closely related to the ability to maintain attention and control responses. Third, cognitive control processes, but not the ability to filter irrelevant information, determine individual differences in working memory.

We relate the identified factor of attention control to the general controlling factor of cognitive control in the Miyake and Friedman model, which is associated with the ability to maintain attention on goals [43,44]. In support of this conclusion, the efficiency of maintenance of attention on the task determines accuracy in the antisaccade task [24,45]. Additionally, the visual arrays task by its nature requires the use of visual attention [46], which is also confirmed by the relationship between capacity k and the ability to actively suppress distractors [47]. The selective visual array task showed a high correlation with the antisaccade task and had a high weight in the total attention control factor of all tests in the study of Draheim et al. [30]. Similarly, Martin et al. [48] showed that, at least in the selective version of the task, performance in visual arrays tasks is better explained by the attention control account. Perhaps, disengagement from the previous trial and maintenance of attention in preparation for the next trial have a greater impact on performance than working memory storage capacity in the visual arrays task. The model with the change detection task loading on attention control rather than on working memory factor showed better convergence in the current study, confirming that change detection tasks are not pure measures of visual working memory storage capacity.

As expected, the attention control factor was associated with the capacity of working memory. The factor loads were in fair agreement with the results obtained before [12,13,30], and the correlation between the factor of attention control and the factor of working memory numerically replicated the results of existing models taking into account the confidence interval (r = 0.69, ci [0.46–0.92], Figure 1).

The identified factor of selective attention possibly reflects the ability to filter irrelevant information and counteract distractors. We can rule out that Stroop and visual search tasks are a reflection of reaction time and associated information processing speed because we used difference scores (RT) in both of these tasks. Visual search, which contributes the most to this factor, is the classic task of counteracting distractor information [27,49]. Such indicators as mean reaction time in a visual search task and the increase in reaction time with an increasing number of distractors have been used in many studies to elucidate individual differences [10,26,50,51,52].

We attributed filtering irrelevant information and counteracting distractors abilities to selective attention, but these functions are frequently associated with attention control [44,53,54,55]. Although related, selective attention and attention control constituted two separate factors in our study. The relationship between attention control and the ability to suppress distractors can be explained by the ability to maintain attention on the target: a stronger top-down activation—the proposed mechanism of cognitive control—leads to a faster correct response [55,56,57]. Alternatively, in the antisaccade task, selective attention may affect sustained activation of the target location, while not interfering with the process of conflict resolution [58].

The relationship between the factor of selective attention and working memory was not confirmed in the present study. Although a number of studies have shown a relationship between working memory performance and visual search [59,60,61,62], this relationship can be explained by the general mechanism of cognitive control. Differences between participants with high and low working memory capacity were observed for tasks that required the retention of a cue where the target was located [63], while no such effect was found for prototypical visual search [64]. Individual differences in working memory can be explained by the ability to filter out distractors [47,65]. However, previous studies employed tasks with physically salient distractors. As there is no stimulus that automatically captures attention in our version of the visual search task, the inhibitory mechanism that suppresses salient stimuli is possibly not active in our study. Perhaps, the ability to counteract attention capture is distinct from the ability to search among stimuli with similar saliency, and the general ability to counteract distractor interference only affects working memory capacity measures in tasks involving salient distractors [66].

The results of the Stroop task are also worth separate consideration. In many studies, the correlation between the Stroop effect (RT difference in ms) from different versions of the task and working memory was small to moderate: r = 0.14 [11], r = 0.20 [14], r = 0.11 [67], r = 0.29 [12], r= 0.24 [68], r = 0.20 [8], r = 0.25 [69]. However, some studies have shown an increase in association with the effect observed when performing a task with a large number of incongruent trials [9,70]. Our results confirmed the correlation between the Stroop task with 50% congruent trials and the operation span, but no increase in correlation was obtained for the task with predominantly incongruent samples. In addition, the Stroop task with 50% congruency, unlike other variants, significantly loaded on both factors—selective attention and attention control. Thus, we hypothesize that differences in Stroop task performance are related to both the ability to maintain attention on the task and the ability to counteract distractor interference. Stroop task and flanker task scores constituted a separate attention control factor in one study [30] but in another study [15], flanker task indicators were derived as a separate factor, and the Stroop test loaded the same factor with the antisaccade test. These results may support our findings that individual differences in Stroop task may be equally strongly related to different types of attention: selective attention and attention control.

## 5. Conclusions

In this study, we examined the relationship between working memory capacity and attention. Our analysis revealed that the best model to describe the data includes working memory capacity, attention control, and selective attention. The results indicate that the processes involved in maintaining focus on a goal and counteracting interference from distractors can be considered distinct factors. Additionally, individual differences in working memory appear to be determined solely by the ability to maintain attention on a task, rather than the ability to filter out distractors.

## Figures and Tables

**Table 1:** Descriptive statistics.

Task	M (SD)	Skew	Kurtosis	Reliability
Before outliers’ exclusion (N = 116)				
Operation span performance	0.71 (0.18)	−0.98	3.84	0.76
Change detection k_c_ average	2.42 (0.54)	−0.17	2.71	0.78
Digit span simple	7.33 (1.28)	0.01	0.39	–
Digit span adaptive	6.72 (1.29)	0.85	1.66	0.76
Stroop effect proactive control (25% congruent)	−0.14 (0.24)	−2.00	6.29	0.48
Stroop effect reactive control (75% congruent)	−0.20 (0.22)	−0.87	4.41	0.34
Stroop effect standard (50% congruent)	−0.14 (0.18)	−1.53	8.82	0.35
Antisaccade accuracy	0.91 (0.07)	−0.98	3.84	0.77
Visual search difference	1.32 (0.53)	1.71	5.17	0.62
Visual search reaction time	2.09 (0.53)	2.30	12.17	0.96
After outliers’ exclusion (N = 111)				
Operation span performance	0.70 (0.18)	−0.57	−0.06	0.75
Change detection k_c_ average	2.38 (0.53)	−0.18	−0.25	0.99
Digit span simple	7.28 (1.28)	0.01	0.39	–
Digit span adaptive	6.72 (1.30)	0.85	1.66	0.76
Stroop effect proactive control (25% congruent)	−0.15 (0.24)	0.09	1.88	0.48
Stroop effect reactive control (75% congruent)	−0.19 (0.22)	−0.43	−0.02	0.35
Stroop effect standard (50% congruent)	−0.15 (0.19)	−0.60	0.80	0.34
Antisaccade accuracy	0.91 (0.07)	−0.73	−0.08	0.77
Visual search difference	1.33 (0.54)	0.00	1.15	0.65
Visual search reaction time	1.67 (0.30)	0.78	0.71	0.99

**Table 2:** Correlation matrix.

	OSA	CD k_c_	DS	DSA	SPE	SRE	SSE	ASA	VSD	VSRT
OSA	1.00	0.22	0.25	**0.48** *	0.12	−0.02	0.23	0.29	−0.03	−0.07
CD k_c_		1.00	0.25	**0.34** *	0.09	0.09	**0.35** *	**0.34** *	−0.11	−0.12
DS			1.00	**0.59** *	0.03	0.06	0.01	**0.35** *	−0.01	−0.07
DSA				1.00	0.15	0.07	0.17	**0.35** *	−0.08	−0.09
SPE					1.00	0.14	−0.07	−0.07	−0.07	−0.09
SRE						1.00	0.13	0.00	−0.15	−0.15
SSE							1.00	0.17	−0.22	−0.24
ASA								1.00	−0.16	−0.25
VSD									1.00	**0.78** *
VSRT										1.00

Notes: OSA—operation span accuracy, DSS—digit span simple (see description of the tasks used), DSA—digit span adaptive, CD k_c_—change detection K-average, ASA—antisaccade accuracy, SPE—proactive Stroop effect, SRE—reactive Stroop effect, SSE—standard Stroop effect, VSRT—visual search reaction time, VSD—visual search difference. * significant Pearson correlation with *p* < 0.05.

**Table 3:** Results of exploratory factor analysis.

	F1	F2	F3
Operation span accuracy	**0.477**	−0.002	0.264
Change detection k_c_ average	0.353	−0.052	**0.427**
Digit span simple	**0.717**	−0.013	−0.054
Digit span adaptive	**0.823**	−0.008	0.156
Stroop effect standard (50% congruent)	0.061	−0.167	**0.741**
Antisaccade accuracy	**0.446**	−0.199	0.173
Visual search difference	−0.039	**0.769**	−0.127
Visual search reaction time	−0.084	**0.989**	−0.098

**Table 4:** Fit indices of CFA models.

Models	*χ* ^2^	*df*	*χ* ^2^ */df*	*p*	RMSEA		SRMR	NNFI	CFI	AIC
					est.	*p*				
Model 1	50.9	19	3.11	**<0.001**	0.135	0.003	0.137	0.800	0.864	602.7
Model 2	32.3	17	1.90	**0.014**	0.088	0.089	0.086	0.914	0.948	580.0
Model 3	23.7	17	1.40	0.127	0.058	0.364	0.061	0.962	0.977	571.4
Model 4	16.9	16	1.06	0.390	0.022	0.670	0.041	0.994	0.997	566.6
Model EFA	37.7	17	2.22	**0.003**	0.102	0.029	0.102	0.884	0.930	585.3

## Data Availability

The data are available upon request from the corresponding author.
